# Supplementary material for: Inter-arm difference in systolic blood pressure: Prevalence and associated factors in an African population
Source: PLoS One. 2022 Aug 31;17(8):e0272619. doi: 10.1371/journal.pone.0272619 (PMC9432703; doi:10.1371/journal.pone.0272619)
Supplement: S1 File — (DOCX) [file pone.0272619.s001.docx]

**Supplement 1**


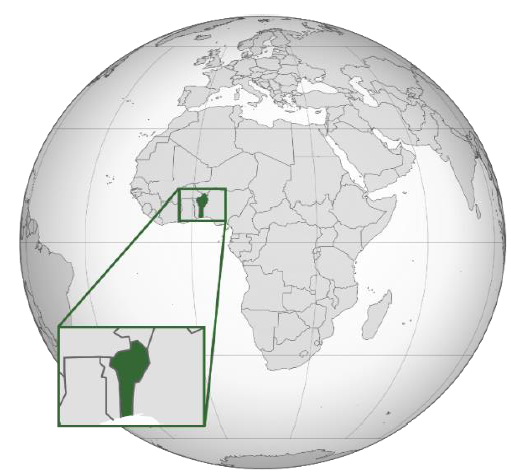

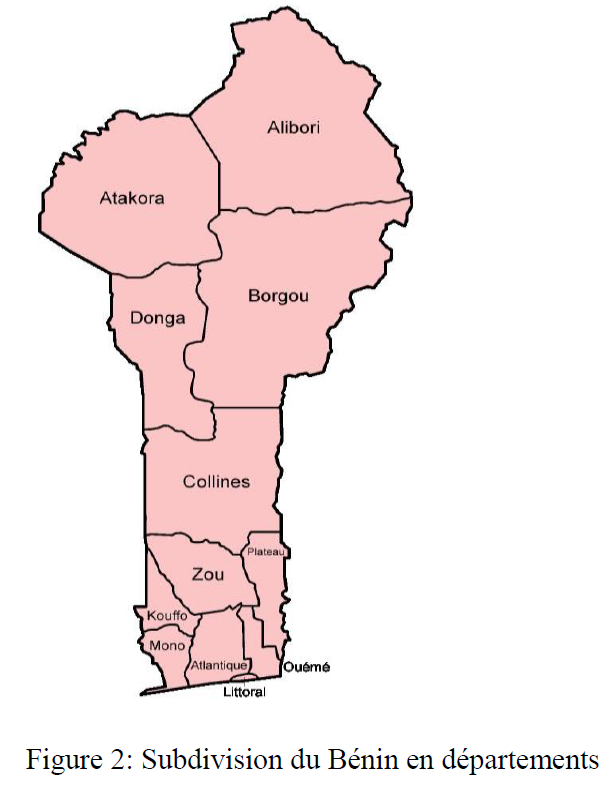


Benin's position in the world

<https://fr.wikipedia.org/wiki/B%C3%A9nin>

Tanve

Supplement 1 : Subdivision of Benin into departments and geographical location of the study area (Tanve area) in Benin.
