# Supplementary material for: Inter-arm difference in systolic blood pressure: Prevalence and associated factors in an African population
Source: PLoS One. 2022 Aug 31;17(8):e0272619. doi: 10.1371/journal.pone.0272619 (PMC9432703; doi:10.1371/journal.pone.0272619)
Supplement: S2 File — (DOCX) [file pone.0272619.s002.docx]

**Supplement 2**: Factors associated with systolic inter-arm blood pressure difference ≥ 15 mmHg, univariate and multivariate analysis, sensitivity analysis, TAHES Study, Benin 2020

|  | Univariate analysis | | Multivariate analysis | |
| --- | --- | --- | --- | --- |
|  | **Crude OR [CI 95%]** | **p-value^Ⱡ^** | **Adjusted OR [CI 95%]** | **p-value^Ⱡ^** |
| Age (per 10 years) | 1.24 [1.11-1.38] | < 0.001 | 1.07 [0.93-1.22] | 0.371 |
| Gender |  |  |  |  |
| Female (vs. male) | 1.11 [0.76-1.63] | 0.588 | 0.99 [0.64-1.54] | 0.979 |
| Education levels |  |  |  |  |
| Illiterate | 1 | < 0.001 | 1 |  |
| Less than primary level | 0.29 [0.13-0.63] |  | 0.37 [0.16-0.82] | 0.003* |
| Primary level and above | 1.15 [0.71-1.86] |  | 1.47 [0.85-2.56] |  |
| Marital status |  |  |  |  |
| Single, widowed or divorced (vs. in couple) | 2.01 [1.31-3.09] | 0.001 | 1.37 [0.82-2.29] | 0.231 |
| Occupation |  |  |  |  |
| Small self-employed without trade register | 1 | 0.36 |  |  |
| Independent farmer/contractor | 1.14 [0.69-1.87] |  |  |  |
| Small business employee/farm worker | 0.50 [0.21-1.16] |  |  |  |
| Private employee or official worker | 0.86 [0.26-2.84] |  |  |  |
| Retired/unemployed/other/student/apprentice | 1.20 [0.68-2.12] |  |  |  |
| Monthly income ($US) |  |  |  |  |
| < 68 | 1 | 0.597 |  |  |
| 68-117 | 1.24 [0.80-1.91] |  |  |  |
| ≥ 117 | 0.99 [0.60-1.64] |  |  |  |
| Tobacco smoking (vs non-smokers) | 1.62 [0.79-3.34] | 0.191 | 1.38 [0.64-2.97] | 0.412 |
| Low intake of fruit & vegetable (Yes vs No) | 0.86 [0.59-1.24] | 0.416 |  |  |
| Sedentarity behavior (Yes vs No) | 1.20 [0.68-2.12] | 0.523 |  |  |
| Alcohol consumption last 30 days (Yes vs No) | 1.11 [0.76-1.61] | 0.584 |  |  |
| BMI (Kg/m^2^) |  |  |  |  |
| Normal | 1 |  |  |  |
| Underweight | 1.17 [0.68-2.03] | 0.221 |  |  |
| Overweight | 1.15 [0.70-1.90] |  |  |  |
| Obesity | 1.89 [1.07-3.35] |  |  |  |
| Hypertension (Yes vs No) | 3.41 [2.32-5.01] | < 0.001 | 2.95 [1.96-4.43] | < 0.001* |
| Diabetes (Yes vs No) | 2.03 [0.94-4.41] | 0.072 | 1.7 [0.76-3.79] | 0.197 |
| Anxiety (Yes vs No) | 1.18 [0.74-1.89] | 0.487 |  |  |
| Depression (Yes vs No) | 1.29 [0.89-1.89] | 0.184 | 1.2 [0.81-1.77] | 0.37 |
| Proteinuria (Yes vs No) | 0.88 [0.31-2.49] | 0.815 |  |  |

OR: Odd ratio ^Ⱡ:^ p value of Wald test for binary variables or likelihood test for categorical variables with more than two modalities

*: statistically significant
