# Supplementary material for: Inter-arm difference in systolic blood pressure: Prevalence and associated factors in an African population
Source: PLoS One. 2022 Aug 31;17(8):e0272619. doi: 10.1371/journal.pone.0272619 (PMC9432703; doi:10.1371/journal.pone.0272619)
Supplement: S3 File — (PDF) [file pone.0272619.s003.pdf]

OUTIL TAHES, ENQUETE D'INCLUSION / VISITE ANNUELLE DE SUIVI

	**A. Identification enquêteurs et répondants**	
**1.**	Identification de l'enquêteur	Equipe 1 / Equipe 2 / Equipe 3 / Equipe 4 / Equipe 5 / Equipe 6 / Equipe 7 / Equipe 8
**2.**	Date et heure de l'entretien (à laquelle le questionnaire a été rempli)	└─┴─┘ └─┴─┘ └─┴─┴─┴─┘ JJ/MM/AAAA └─┴─┘ └─┴─┘ hrs mins
**3.**	Numéro d'identification du répondant	└─┴─┴─┴─┴─┴─┴─┴─┘
**4.**	Type répondant	Ancien / Nouveau
**5.**	Le consentement a été obtenu (oral ou écrit)	Oui / Non
**6.**	Langue de l'entretien	Français / Fon / Autre
**6.1.**	Si autre langue, précisé	…………………………………………………………
	**B. Informations démographiques**	
**7.**	Sexe	Homme / Femme
**7.1.**	Si sexe féminin, gestité (nombre de grossesses)	└─┴─┘
**7.2.**	7-2.Si sexe féminin, parité (nombre d'enfants vivant)	└─┴─┘
**7.3.**	Etes-vous actuellement enceinte ?	Oui / Non
**7.4.**	Si oui de combien de semaines d'aménorrhée	…………………………………………………………
**7.5.**	Avez-vous déjà eu de l'HTA pendant la grossesse?	Oui / Non
**7.6.**	Avez-vous déjà pris des médicaments pour l'HTA pendant la grossesse?	Oui / Non
**7.7.**	Année de fin de la 1ère grossesse (accouchement, avortement...)	…………………………………………………………
**7.8.**	Année de fin la dernière grossesse (accouchement, avortement...)	…………………………………………………………
**7.9.**	Lieu d'accouchement lors de la dernière grossesse (si première grossesse, lieu d'accouchement envisagé)	Domicile / Maternité périphérique (1ère intention) / Centre de référence
**8.**	Je souhaiterais connaître votre âge. Avez-vous une pièce d'identité? (Acte de naissance, CNI, Passeport, Permis, LEPI, etc...)	Oui / Non
	Si oui, notez la date naissance	└─┴─┘ └─┴─┘ └─┴─┴─┴─┘ JJ/MM/AAAA
	Si absence document d'identité, quel est votre âge?	└─┴─┴─┘
	Comment l'âge a été renseigné?	Sur déclaration de l'enquêté / Par estimation à l'aide d'outil
**9.**	Depuis quand vivez-vous à Tanvè	└─┴─┘ └─┴─┘ └─┴─┴─┴─┘ JJ/MM/AAAA
**10.**	En tout, combien d'années avez-vous passé à l'école ou à suivre une formation à plein temps (sans la pré-scolarité)	└─┴─┘ Années
**10.1.**	Quel est le <u>plus haut niveau</u> d'instruction que vous ayez atteint ?	Aucune instruction / primaire sans CEP / A le CEP / A le BEPC / A le BAC / A un diplôme après le bac
**11.**	A quel groupe ethnique appartenez-vous ?	Fon Autre (Préciser)……………………
**12.**	Quelle est votre religion ?	Catholique/ Autre chrétienne / Islam / Animiste/ Autre (Préciser)……………………
**13.**	Quel est votre statut matrimonial ?	Marié ou en couple / Célibataire / Veuf ou divorcé
**13.1.**	Si marié ou en couple, foyer polygame?	Oui /Non
**14.**	Etes-vous alphabétisé dans une langue locale	Oui /Non
**14.1.**	Si oui, quelle langue?	……………………………………

**15.**	Laquelle des catégories suivantes décrit le mieux votre activité professionnelle principale ces 12 derniers mois ?	Agriculteur / Ouvrier agricole / Revendeur/artisan/taxi-moto / Employé de petit commerce / Employé du privé ou fonctionnaire / Elève, étudiant, apprenti / Commerçant et entrepreneur avec régistre de commerce / Retraité / Sans emploi / Autre (précisez) …………………………………………………….
**16.**	Votre activité principale est- elle permanente ou saisonnière ?	Permanente / Saisonnière
**17.**	Combien de personnes vivent dans votre ménage ?	⊔⊔
**18.**	En prenant comme référence les 12 derniers mois, pouvez-vous estimer le revenu mensuel moyen de votre ménage ?	≤ 40 000 / 40 001-70 000 / 70 001-105 000 / 105 001-140 001 / 140 000-175 000 / 175 001-205 000 / > 205000
**19.**	**Paramètres du niveau socio-économique**	
**19.1.**	Quelle est la source d'eau principale utilisée par votre ménage	Fontaine, robinet / Rivière, mare, eau de pluie, puit
**19.1.1.**	Si fontaine ou robinet, est-ce situé dans votre maison? (Payez-vous une facture SONEB? ou avez-vous une fontaine chez vous?)	Oui /Non
**19.2.**	A quelle distance (trajet - aller) se situe cette source d'eau	< ou = 2 km / > 2 km
**19.3.**	Disposez-vous dans l'habitation ou à proximité de latrines à fosses ou latrines à chasses?	Oui /Non
**19.4**	Une personne dans votre ménage possède-t-elle une télévision ?	Oui /Non
**19.5**	Une personne dans votre ménage possède-t-elle une radio ?	Oui /Non
**19.6**	Une personne dans votre ménage possède-t-elle une voiture ?	Oui /Non
**19.7**	Une personne dans votre ménage possède-t-elle une moto ?	Oui /Non
**19.8.**	Une personne dans votre ménage possède-t-elle un vélo ?	Oui /Non
**19.9.**	Avez-vous un salon meublé? (canapé et autres mobiliers en bois et/ou en mousse)	Oui /Non
**19.10.**	Une personne dans votre ménage possède-t-elle un compte épargne, bancaire ou une épargne tontine ?	Oui /Non
**19.11.**	Une personne dans votre ménage possède-t-elle un téléphone portable ?	Oui /Non
**19.12.**	Une personne dans votre ménage possède-t-elle, une maison, une terre ou une portion de terre?	Oui /Non
**19.13.**	Observez les revêtements principaux des murs, du toit et du sol, et précisez :	Mur dur ou semi-dur : terre, pierre, brique / mur en matériau rudimentaire : paille, bois, planche<br>Toit moderne ou naturel : tôle, tuile, dalle, paille / toit précaire: palme, bambous, bois, planche<br>Sol dur : sable, terre, ciment, parquet, carreaux / sol précaire: palme, bois, planche
	**C. STEP1**	
	**C1. CONSOMMATION TABAC**	
**20.**	Fumez-vous des produits à base de tabac tels que cigarettes, cigares ou pipes ?	Oui /Non
**20.1.**	Si oui, fumez-vous quotidiennement?	Oui /Non
**20.2.**	Depuis quand fumez-vous quotidiennement?	⊔⊔ ⊔⊔ ⊔⊔⊔⊔ JJ/MM/AAAA
**20.3.**	Combien de cigarettes fumez-vous par jour?	⊔⊔
**21.**	Consommez-vous du tabac à priser ou à mâcher?	oui<12 mois / non, jamais / non,>12 mois
**21.1.**	Consommez-vous quotidiennement ces produits?	Oui /Non

**21.2.**	Combien de fois par jour en consommez-vous au cours de ces journées?	└─┴─┘
	**C2. CONSOMMATION D'ALCOOL**	
**22.**	Avez-vous consommé une boisson alcoolisée (bière, vin, liqueur, Sodabi, ou Atan) ces 12 derniers mois ?	Oui /Non
**22.1.**	Si Oui, à quelle fréquence avez- vous bu au moins une boisson alcoolisée ?	Quotidiennement / 5-6 jours par semaine / 1-4 jours par semaine / 1-3 jours par mois / moins d'une fois par mois
**22.2.**	Quand vous buvez de l'alcool, combien de verres prenez-vous en moyenne par jour ?	└─┴─┴─┘
**22.3.**	Avez-vous consommé une boisson alcoolisée (bière, vin, liqueur, sodabi, atan) ces 30 derniers jours ?	Oui /Non
**22.4.**	Au cours des 7 derniers jours, quelle quantité de boisson alcoolisée avez-vous pris en moyenne (quelle que soit la boisson alcoolisée) ? Décrire...	……………………………………………………………………………………………… ……………………………………………………………………………………………… ………………………………………………………………………………………………
**22.4.1**.	Equivalents à .... verres standards	└─┴─┴─┘
	**C3. HYGIÈNE ALIMENTAIRE**	
**23.**	Habituellement, combien de jours par semaine consommez-vous des fruits ?	…………………………………
**23.1.**	Combien de portions de fruits mangez-vous lors d'une de ces journées ?	└─┴─┘
**24.**	Habituellement, combien de jours par semaine consommez-vous des légumes ?	…………………………………
**24.1.**	Combien de portions de légumes mangez-vous lors d'une de ces journées ?	└─┴─┘
**25.**	Ajoutez-vous du sel de cuisine aux aliments lors de leur cuisson?	Jamais / quelquefois / souvent / toujours
**25.1.**	Ajoutez-vous du sel de cuisine aux aliments au cours des repas?	Jamais / quelquefois / souvent / toujours
**25.2.**	Utilisez-vous du cube, arome ou des produits en boites de conserves (tomates, sardines, thon, autres…) ?	Jamais / quelquefois / souvent / toujours
**25.4.**	Combien de fois par jour mangez-vous des plats cuisinés à la maison?	Jamais / quelquefois / souvent / toujours
	**C4. COMPORTEMENT SEDENTAIRE**	
**26.**	Combien de temps passez-vous en position assise ou couchée lors d'une journée habituelle ? (en heures, n'inclut pas le temps de sommeil)	└─┴─┘
**26.1.**	Pouvez-vous décrire votre activité de journée ?	Assis la plupart du temps / Debout ou petits déplacements sans soulèvement de charge, ni effort physique important / Debout, marche prolongée, travail physique important (Soulèvement de charge, montée plus de trois étages plusieurs fois par jours…)
	**C5. ACTIVITÉ PHYSIQUE**	
**27.**	Habituellement combien de jours par semaine pratiquez- vous une activité physique modérée d'au moins 30 minutes ou intense d'au moins 15 minutes dans le cadre de vos activités ou vos loisirs?	< 3 jours / 3 à 5 jours / 6 à 7 jours
**27.1.**	Combien de minutes en moyenne marchez-vous par jour (déplacement au travail non inclus)?	└─┴─┴─┘

**27.2.**	Combien de minutes en moyenne allez-vous à vélo par jour (déplacement au travail non inclus)?	└──┴──┴──┘
	**C6. ANTÉCÉDENTS (HTA/DIABETE/MCV/IRC/AUTRES)**	
**28.**	Quand est-ce que votre Pression artérielle a été prise pour la dernière fois par un agent de santé ?	< 12 mois / 1-5 ans / > 5 ans / jamais
**29.**	Au cours des 24 derniers mois, est-ce qu'un agent de santé vous a dit que vous aviez une pression artérielle élevée ou que vous souffriez d'hypertension?	Oui /Non
**30.**	Recevez- vous actuellement des médicaments prescrits par un agent de santé pour une pression artérielle élevée ?	Oui /Non
**31.**	Suivez- vous actuellement des prescriptions d'un agent de santé tel que un régime spécial pour perdre du poids, arrêter de fumer, commencer une activité physique pour une pression artérielle élevée ?	Oui /Non
**31.1.**	Au cours des 24 derniers mois, avez-vous vu un guérisseur traditionnel pour le traitement d'une HTA?	Oui /Non
**32.**	Quand est-ce que votre glycémie a été prise pour la dernière fois par un agent de santé ?	< 12 mois / 1-5 ans / > 5 ans / jamais
**33.**	Est-ce qu'un professionnel de santé vous a déjà dit que vous aviez une glycémie élevée ou du diabète au cours de ces 24 derniers mois ?	Oui /Non
**34.**	Au cours des 3 derniers mois, ressentez-vous:	
**34.1.**	Une augmentation importante de la fréquence ou quantité des urines?	Oui /Non
**34.2.**	Une soif intense vous imposant de boire fréquemment?	Oui /Non
**34.3.**	Une fatigue inhabituelle, une prise de poids ou amaigrissement rapides?	Oui /Non
**34.4.**	Aucun de ces signes	Oui /Non
**35.**	Recevez- vous actuellement les traitements suivants prescrits par un professionnel de santé pour le diabète, et/ou les conseils suivants ?	
**35.1.**	Insuline	Oui /Non
**35.2.**	Médicament par voie orale pris ces 2 dernières semaines	Oui /Non
**35.3.**	Régime, conseil ou traitement pour perdre du poids, arrêter de fumer, pratiquer une activité physique?	Oui /Non
**35.4.**	Aucun traitement de diabète	Oui /Non
**36.**	Au cours des 24 derniers mois avez-vous vu un guérisseur traditionnel pour le diabète ?	Oui /Non
**37.**	Est-ce qu'un professionnel de santé vous a déjà diagnostiqué une affection cardiovasculaire ou neurologique ?	Oui /Non
**37.1.**	Insuffisance cardiaque	Oui /Non
**37.2.**	Accident vasculaire cérébral	Oui /Non
**37.3.**	Angine de poitrine	Oui /Non
**37.4.**	Infarctus du myocarde	Oui /Non
**37.5.**	Maladie artérielle périphérique	Oui /Non

**38.**	Est-ce qu'un professionnel de santé vous a déjà diagnostiqué une autre affection chronique ? cancer, Hépatite chronique, troubles psychiatriques?	Oui /Non
**38.1.**	Si oui, préciser le ou les affections	
**38.2.**	Est-ce qu'un professionnel de santé vous a déjà diagnostiqué une hématurie (sang dans les urines)?	Oui /Non
**38.3.**	Est-ce qu'un professionnel de santé vous a déjà diagnostiqué une albuminurie ? (Présence de sel dans les urines)	Oui /Non
**38.4.**	Est-ce qu'un professionnel de santé vous a déjà diagnostiqué une infection urinaire ? (Brûlures lors de l'émission des urines)	Oui /Non
**38.5.**	Est-ce qu'un professionnel de santé vous a déjà diagnostiqué une insuffisance rénale ?	Oui /Non
	**C6.1 Remède traditionnel / Automédication**	
**39.**	Prenez-vous un remède traditionnel à base d'écorces, de racines ou de feuilles...	
39.a.	Contre l'Hypertension artérielle ?	Oui /Non
39.a.1.	Si oui la(les)quelle (s) remède pour HTA? (nom vernaculaire)	Plante 1 / Plante 2 / Plante 3 / Plante 4
39.a.2.	Si oui, à quelle fréquence prenez-vous ces plantes médicinales ?	Tous les jours / Quelques fois par semaine / Quelques fois par mois / Quelques fois par an
39.a.3.	Sur quelle durée utilisez-vous ces plantes médicinales ?	< 1 mois / 1 à 11 mois / 1 à 4 ans / ≥ 5ans
39.b.	Contre le Diabète ?	Oui /Non
39.b.1	Si oui la(les)quelle (s) remède pour le diabète? (nom vernaculaire)	Plante 1 / Plante 2 / Plante 3 / Plante 4
39.b.2.	Si oui, à quelle fréquence prenez-vous ces plantes médicinales ?	Tous les jours / Quelques fois par semaine / Quelques fois par mois / Quelques fois par an
39.b.3.	Sur quelle durée utilisez-vous ces plantes médicinales ?	< 1 mois / 1 à 11 mois / 1 à 4 ans / ≥ 5ans
39.c.	Contre toute autre maladie?	Oui /Non
39.c.1.	Préciser les deux principales autres affections.	Affection 1 / Affection 2 / Affection 3 / Affection 4
39.c.2.	Si oui la(les)quelle (s) remède pour et ? (nom vernaculaire)	Plante 1 / Plante 2 / Plante 3 / Plante 4
39.c.3.	Si oui, à quelle fréquence prenez-vous ces plantes médicinales ?	Tous les jours / Quelques fois par semaine / Quelques fois par mois / Quelques fois par an
39.c.4.	Sur quelle durée utilisez-vous ces plantes médicinales ?	< 1 mois / 1 à 11 mois / 1 à 4 ans / ≥ 5ans
39.d.	Pour la prévention des maladies?	Oui /Non
39.d.1.	Si oui la(les)quelle (s) pour prévention ? (nom vernaculaire)	Plante 1 / Plante 2 / Plante 3 / Plante 4
39.d.2.	Si oui, à quelle fréquence prenez-vous ces plantes médicinales ?	Tous les jours / Quelques fois par semaine / Quelques fois par mois / Quelques fois par an
39.d.3.	Sur quelle durée utilisez-vous ces plantes médicinales ?	< 1 mois / 1 à 11 mois / 1 à 4 ans / ≥ 5ans
39.1.	Faites-vous de l'automédication ?	Oui /Non
39.1.1.	Si oui, quels sont les produits que vous achetez souvent ?	AINS (ibuprofène, Diclofénac, Indocid, Aspirine, Ibucap, Socomol…) / Antalgique (paracétamol, efferalgan ParaFizz, Tramol, Tramadol, …) / Antibiotiques (amoxicilline, métronidazole, Bactrim, Cotrimoxazole, Ciprofloxacine / Autres (à préciser)
39.1.2.	Si oui à quelle fréquence ?	Tous les jours / Quelques fois par semaine / Quelques fois par mois / Quelques fois par an

39.1.3.	Sur quelle durée utilisez-vous ces médicaments ?	< 1 mois / 1 à 11 mois / 1 à 4 ans / ≥ 5ans
	**C8- WHO ROSE Angine Questionnaire**	
**40.**	Ressentez-vous une douleur ou une gêne dans la poitrine quand vous marchez ?	Oui /Non
**41.**	Ressentez-vous cette douleur quand vous montez une côte ou quand vous marchez vite ?	Oui /Non
**42.**	La ressentez-vous même quand vous marchez d'un pas normal sur un terrain plat ?	Oui /Non
**43.**	Lorsque la douleur survient à la marche, que faites- vous ?	Vous ralentissez / vous vous arrêtez / vous continuez ce que vous faites
**44.**	Que devient la douleur quand vous vous arrêtez ?	Elle persiste plus de 10 mn / elle disparaît habituellement en 10 mn ou moins /
**45.**	Où ressentez-vous cette douleur ou gène ? à indiquer sur image 1	Poitrine précordiale droite / Poitrine précordiale gauche / Poitrine retrosternale / Epigastrique / Abdomen (hors épigastre) / Bras droit / Bras gauche
**46.**	Avez-vous déjà ressenti une fois une douleur thoracique ou une gêne atroce dans la poitrine au repos ayant duré 30 minutes ou plus ?	Oui /Non
	**C9- Edimbourg Claudication Questionnaire**	
**47.**	Ressentez-vous une douleur ou une gêne dans une jambe quand vous marchez ?	Oui /Non
**48.**	Cette douleur commence-t- elle parfois à se manifester quand vous êtes debout immobile ou assis ?	Oui /Non
**49.**	Ressentez-vous cette douleur quand vous montez une côte ou quand vous marchez vite ?	Oui /Non
**50.**	Le ressentez-vous-même quand vous marchez d'un pas normal sur un terrain plat ?	Oui /Non
**51.**	Que devient la douleur quand vous vous arrêtez ?	Elle persiste plus de 10 mn / elle disparaît habituellement en 10 mn ou moins
**52.**	Où ressentez-vous cette douleur ou gène ? à indiquer sur image 2	Bassin / Cuisse / Genou / Mollet / Jambe (autre partie que mollet) / Cheville / Plante du pied / Dos du pied
	**C10- Questions Insuffisance Cardiaque**	
**53.**	Avez-vous déjà ressenti ou ressentez-vous un essoufflement (+/- fatigue, toux ou palpitations) apparaissant lors des efforts importants (montée de deux escaliers ou plus, d'une côte par exemples ?)	Oui /Non
**54.**	Avez-vous déjà ressenti ou ressentez-vous un essoufflement (+/- fatigue, toux ou palpitations) apparaissant lors des efforts importants (montée d'un escalier par exemple ?)	Oui /Non
**55.**	Avez-vous déjà ressenti ou ressentez-vous un essoufflement (+/- fatigue, toux ou palpitations) apparaissant lors des efforts minimes, ordinaires tels que se laver, ou de petits déplacements?	Oui /Non
**56.**	Avez-vous déjà ressenti ou ressentez-vous un essoufflement (+/- fatigue, toux ou palpitations) au repos, s'aggravant en position couchée ou la nuit?	Oui /Non
56.1.	Si oui, l'essoufflement était-il associé à des œdèmes des membres inférieurs?	Oui /Non
**57.**	Ces symptômes ont t'ils disparu spontanément ou sous traitement ?	Persistant / disparu spontanément / disparu sous traitement
**58.**	Aviez-vous consulté dans un centre de santé ou auprès d'un guérisseur ?	Oui / non / disparu sous traitement

**58.1.**	Si oui, préciser le nom du centre ou du guérisseur	
**59.**	Quand avez- vous ressenti ces symptômes (l'épisode le plus récent) ?	└─┴─┘ └─┴─┘ └─┴─┴─┴─┘ JJ/MM/AAAA
	**C11- AVC**	
**60.**	Avez-vous déjà eu de façon soudaine et pendant au moins 24 heures l'un des signes suivants :	Aucun
60.1.	Une perte de sensibilité ou une sensation anormale de vos bras ou vos jambes ?	Oui /Non
60.2.	Une faiblesse ou une paralysie d'un côté de votre corps, d'un de vos bras, d'une de vos jambes, ou de vos deux jambes ?	Oui /Non
60.3.	une paralysie d'un côté de la face?	Oui /Non
60.4.	Des difficultés à comprendre ce que les gens vous disent, à parler, à lire ou à écrire?	Oui /Non
60.5.	Une perte de la vision d'un œil ou des deux yeux ou de la moitié de la vision?	Oui /Non
**61.**	Avez-vous eu un des problèmes suivants accompagnant l'un des symptômes suscités : tremblement incontrôlé des membres, Maux de tête, Vertiges, Perte de Connaissance, Convulsions?	Oui /Non
	**C12- Anxiété (9 items +1)**	
**62.**	Vous sentez-vous tendu (e) ou à bout ?	Oui /Non
**63.**	Vous faites-vous beaucoup de soucis ?	Oui /Non
**64.**	Vous êtes- vous senti (e) irritable ?	Oui /Non
**65.**	Avez-vous eu des difficultés à vous détendre ?	Oui /Non
**66.**	Dormez-vous mal ?	Oui /Non
**67.**	Avez-vous des maux de tête ou de la nuque ?	Oui /Non
**68.**	Avez-vous eu un des problèmes suivants : Tremblements, picotement, sensation de déséquilibre, sueurs, diarrhées, envie fréquente d'uriner ?	Oui /Non
**69.**	Vous faites- vous du souci pour votre santé ?	Oui /Non
**70.**	Avez-vous de la peine pour vous endormir ?	Oui /Non
**71.**	Combien de cotisation pour funérailles avez-vous fait les 12 derniers mois? (en dehors des "enveloppes"...)	└─┴─┴─┘
	**C13- Dépression (9 items)**	
**72.**	Manquez-vous d'énergie?	Oui /Non
**73.**	Avez-vous perdu confiance en vous?	Oui /Non
**74.**	Avez-vous perdu l'intérêt pour vos activités habituelles ?	Oui /Non
**75.**	Vous est-il arrivé de vous sentir sans espoir ?	Oui /Non
**76.**	Avez-vous eu des difficultés à vous concentrer?	Oui /Non
**77.**	Avez-vous perdu du poids en raison d'une perte d'appétit ?	Oui /Non
**78.**	Vous êtes- vous réveillé(e) plus tôt que d'habitude ?	Oui /Non
**79.**	Vous êtes- vous senti(e) ralenti(e) ?	Oui /Non
**80.**	Avez-vous eu tendance à vous sentir moins bien le matin ?	Oui /Non
	**D. STEP 2**	
**81.**	PAS bras gauche (mm Hg)	……………………………………

**82.**	PAD bras gauche (mm Hg)	....
**83.**	Pouls gauche	....
**81'.**	PAS bras gauche 2 (mm Hg)	....
**82'.**	PAD bras gauche 2 (mm Hg)	....
**83'.**	Pouls gauche 2	....
**81''.**	PAS bras gauche 3 (mm Hg)	....
**82''.**	PAD bras gauche 3 (mm Hg)	....
**83''.**	Pouls gauche 3	....
**84.**	PAS bras droit (mm Hg)	....
**85.**	PAD bras droit (mm Hg)	....
**86.**	Pouls bras droit	....
**84'.**	PAS bras droit 2 (mm Hg)	....
**85'.**	PAD bras droit 2 (mm Hg)	....
**86'.**	Pouls bras droit 2	....
**84''.**	PAS bras droit 3 (mm Hg)	....
**85''.**	PAD bras droit 3 (mm Hg)	....
**86''.**	Pouls bras droit 3	....
**87.**	Poids (Kg)	....
**88.**	Taille (cm)	....
**89.**	Tour de taille (cm)	....
**90.**	Tour de hanche (cm)	....
**91.**	Dyspnée (au repos ou moindre effort)	Oui /Non
**92.**	Amputation membre ou portion membre (d'origine non traumatique)	Oui /Non Si oui, préciser siège
**93.**	Œdèmes des membres inférieurs	Non / oui, bilatéraux / oui, unilatéral droit / oui, unilatéral gauche
**94.**	Si OMI, godet?	Oui /Non
	**E. STEP 3**	
**95.**	Glycémie capillaire à jeun (mg/dl)	
**96.**	Leucocytes	Négatif / Trace / +70 / ++125 / +++500
**97.**	Nitrite	Négatif / Trace / Positif
**98.**	Urobilinogène	0,1 / 1(16) / 2(33) / 4(66) / 8(131)
**99.**	Protéine	Négatif / Trace / +30(0,3) / ++100(1,0) / +++300(3,0) / ++++1000(10)
**100.**	PH	5 / 6 / 6,5 / 7 / 7,5 / 8 / 8,5
**101.**	Sang	Négatif / Trace / +25 / ++80 / +++200 / non hemolysis+10 / non hemolysis++80
**102.**	Cétones	Négatif / +/-5(5.5) / +15(1.5) / ++40(3.9) / +++80(8) / ++++160(16)
**103.**	Bilirubine	Négatif / + / ++ / +++
**104.**	Densité	1.000 / 1.005 / 1.010 / 1.015 / 1.020 / 1.025 / 1.030
**105.**	Glucose NEGATIF	Négatif / +/-100(5.5) / +250(14) / ++500(28) / +++1000(55) / ++++2000(111)
