# Supplementary material for: Inter-arm difference in systolic blood pressure: Prevalence and associated factors in an African population
Source: PLoS One. 2022 Aug 31;17(8):e0272619. doi: 10.1371/journal.pone.0272619 (PMC9432703; doi:10.1371/journal.pone.0272619)
Supplement: S4 File — (PDF) [file pone.0272619.s004.pdf]

WHO STEPS Instrument

(Core and Expanded)

The WHO STEPwise approach to chronic disease risk factor surveillance (STEPS)

World Health Organization
20 Avenue Appia, 1211 Geneva 27, Switzerland

For further information: www.who.int/chp/steps

STEPS Instrument

Overview

Introduction

This is the generic STEPS Instrument which sites/countries will use to develop their tailored instrument. It contains the:

- CORE items (unshaded boxes)
- EXPANDED items (shaded boxes).

Core Items

The Core items for each section ask questions required to calculate basic variables. For example:

- current daily smokers
- mean BMI.

Note: All the core questions should be asked, removing core questions will impact the analysis.

Expanded items

The Expanded items for each section ask more detailed information. Examples include:

- use of smokeless tobacco
- sedentary behaviour.

Guide to the columns

The table below is a brief guide to each of the columns in the Instrument.

Column	Description	Site Tailoring
Number	This question reference number is designed to help interviewers find their place if interrupted.	Renumber the instrument sequentially once the content has been finalized.
Question	Each question is to be read to the participants	• Select sections to use.<br>• Add expanded and optional questions as desired.
Response	This column lists the available response options which the interviewer will be circling or filling in the text boxes. The skip instructions are shown on the right hand side of the responses and should be carefully followed during interviews.	• Add site specific responses for demographic responses (e.g. C6).<br>• Change skip question identifiers from code to question number.
Code	The column is designed to match data from the instrument into the data entry tool, data analysis syntax, data book, and fact sheet.	This should never be changed or removed. The code is used as a general identifier for the data entry and analysis.

Participant Identification Number └─┴─┴─┘ └─┴─┴─┘ └─┴─┴─┘

WHO STEPS Instrument
for Chronic Disease Risk Factor Surveillance

<insert country/site name>

Survey Information

Location and Date		Response	Code
1	Cluster/Centre/Village ID	└─┴─┴─┘	I1
2	Cluster/Centre/Village name		I2
3	Interviewer ID	└─┴─┴─┘	I3
4	Date of completion of the instrument	└─┴─┘ └─┴─┘ └─┴─┴─┴─┘<br>dd mm year	I4

Participant Id Number └─┴─┴─┘ └─┴─┴─┘ └─┴─┴─┘

Consent, Interview Language and Name		Response	Code
5	Consent has been read and obtained	Yes 1<br>No 2 **If NO, END**	I5
6	Interview Language *[Insert Language]*	English 1<br>*[Add others]* 2<br>*[Add others]* 3<br>*[Add others]* 4	I6
7	Time of interview<br>(24 hour clock)	└─┴─┘ : └─┴─┘<br>hrs mins	I7
8	Family Surname		I8
9	First Name		I9
**Additional Information that may be helpful**			
10	Contact phone number where possible		I10

Record and file identification information (I5 to I10) separately from the completed questionnaire.

Step 1 Demographic Information

CORE: Demographic Information

Question		Response	Code
11	Sex (*Record Male / Female as observed)*	Male 1<br>Female 2	C1
12	What is your date of birth?<br>*Don't Know 77 77 7777*	└─┴─┘ └─┴─┘ └─┴─┴─┴─┘ dd mm year *If known, Go to C4*	C2
13	How old are you?	Years └─┴─┘	C3
14	In total, how many years have you spent at school or in full-time study (excluding pre-school)?	Years └─┴─┘	C4

EXPANDED: Demographic Information

15	What is the **highest level of education** you have completed?<br>*[INSERT COUNTRY-SPECIFIC CATEGORIES]*	No formal schooling 1<br>Less than primary school 2<br>Primary school completed 3<br>Secondary school completed 4<br>High school completed 5<br>College/University completed 6<br>Post graduate degree 7<br>Refused 88	C5
16	What is your *[insert relevant ethnic group / racial group / cultural subgroup / others]* **background**?	[*Locally defined]* 1<br>[*Locally defined]* 2<br>[*Locally defined]* 3<br>Refused 88	C6
17	What is your **marital status**?	Never married 1<br>Currently married 2<br>Separated 3<br>Divorced 4<br>Widowed 5<br>Cohabitating 6<br>Refused 88	C7
18	Which of the following best describes your **main work** status over the past 12 months?<br>*[INSERT COUNTRY-SPECIFIC CATEGORIES]*<br>*(USE SHOWCARD)*	Government employee 1<br>Non-government employee 2<br>Self-employed 3<br>Non-paid 4<br>Student 5<br>Homemaker 6<br>Retired 7<br>Unemployed (able to work) 8<br>Unemployed (unable to work) 9<br>Refused 88	C8
19	How many people older than 18 years, including yourself, live in your household?	Number of people └─┴─┘	C9

**EXPANDED: Demographic Information,** Continued				
**Question**		**Response**		**Code**
20	Taking **the past year**, can you tell me what the average earnings of the household have been?<br>*(RECORD ONLY ONE, NOT ALL 3)*	Per week	└─┴─┴─┴─┴─┴─┴─┘ *Go to T1*	C10a
		OR per month	└─┴─┴─┴─┴─┴─┴─┘ *Go to T1*	C10b
		OR per year	└─┴─┴─┴─┴─┴─┴─┘ *Go to T1*	C10c
		Refused	88	C10d
21	If you don't know the amount, can you give an **estimate** of the annual household income if I read some options to you? Is it<br>*[INSERT QUINTILE VALUES IN LOCAL CURRENCY]*<br><br>*(READ OPTIONS)*	≤ Quintile (Q) 1	1	C11
		More than Q 1, ≤ Q 2	2	
		More than Q 2, ≤ Q 3	3	
		More than Q 3, ≤ Q 4	4	
		More than Q 4	5	
		Don't Know	77	
		Refused	88	

Step 1 Behavioural Measurements

**CORE: Tobacco Use**				
Now I am going to ask you some questions about various health behaviours. This includes things like smoking, drinking alcohol, eating fruits and vegetables and physical activity. Let's start with tobacco.				
**Question**		**Response**		**Code**
22	Do you currently smoke any **tobacco products**, such as cigarettes, cigars or pipes? *(USE SHOWCARD)*	Yes	1	T1
		No	2 *If No, go to T6*	
23	Do you currently smoke tobacco products **daily**?	Yes	1	T2
		No	2 *If No, go to T6*	
24	How old were you when you **first started** smoking daily?	Age (years)<br>Don't know 77	└─┴─┘ *If Known, go to T5a*	T3
25	Do you remember how long ago it was?<br><br>*(RECORD ONLY 1, NOT ALL 3)*<br><br>*Don't know 77*	In Years	└─┴─┘ *If Known, go to T5a*	T4a
		OR in Months	└─┴─┘ *If Known, go to T5a*	T4b
		OR in Weeks	└─┴─┘	T4c
26	On average, **how many** of the following do you smoke each day?<br><br>*(RECORD FOR EACH TYPE, USE SHOWCARD)*<br><br>*Don't Know 77*	Manufactured cigarettes	└─┴─┘	T5a
		Hand-rolled cigarettes	└─┴─┘	T5b
		Pipes full of tobacco	└─┴─┘	T5c
		Cigars, cheroots, cigarillos	└─┴─┘	T5d
		Other	└─┴─┘ *If Other, go to T5other, else go to T9*	T5e
		Other (please specify):	└─┴─┴─┴─┴─┴─┘<br>*Go to T9*	T5other

EXPANDED: Tobacco Use

Question		Response	Code
27	In the past, did you **ever** smoke **daily**?	Yes 1<br>No 2 *If No, go to T9*	T6
28	How old were you when you **stopped** smoking **daily**?	Age (years)<br>Don't Know 77 └─┴─┘ *If Known, go to T9*	T7
29	How **long ago** did you stop smoking daily?<br>*(RECORD ONLY 1, NOT ALL 3)*<br>*Don't Know 77*	Years ago └─┴─┘ *If Known, go to T9*	T8a
		OR Months ago └─┴─┘ *If Known, go to T9*	T8b
		OR Weeks ago └─┴─┘	T8c
30	Do you **currently use** any **smokeless tobacco** such as *[snuff, chewing tobacco, betel]*? *(USE SHOWCARD)*	Yes 1<br>No 2 *If No, go to T12*	T9
31	Do you **currently use smokeless tobacco** products **daily?**	Yes 1<br>No 2 *If No, go to T12*	T10
32	On average, how many **times a day** do you use ….<br>*(RECORD FOR EACH TYPE, USE SHOWCARD)*<br>*Don't Know 77*	Snuff, by mouth └─┴─┘	T11a
		Snuff, by nose └─┴─┘	T11b
		Chewing tobacco └─┴─┘	T11c
		Betel, quid └─┴─┘	T11d
		Other └─┴─┘ *If Other, go to T11other, else go to T13*	T11e
		Other (specify) └─┴─┴─┴─┴─┴─┴─┘ *Go to T13*	T11other
33	In the **past**, did you **ever use** smokeless tobacco such as *[snuff, chewing tobacco, or betel]* **daily**?	Yes 1<br>No 2	T12
34	During the past 7 days, on how many days did someone **in your home** smoke when you were present?	Number of days<br>Don't know 77 └─┴─┘	T13
35	During the past 7 days, on how many days did someone smoke in closed areas **in your workplace** (in the building, in a work area or a specific office) when you were present?	Number of days<br>Don't know or don't work in a closed area 77 └─┴─┘	T14

CORE: Alcohol Consumption

The next questions ask about the consumption of alcohol.

Question		Response	Code
36	Have you **ever** consumed an alcoholic drink such as beer, wine, spirits, fermented cider or *[add other local examples]*?<br>*(USE SHOWCARD OR SHOW EXAMPLES)*	Yes 1<br>No 2 *If No, go to D1*	A1a
37	Have you consumed an alcoholic drink within the **past 12 months**?	Yes 1<br>No 2 *If No, go to D1*	A1b
38	During the past 12 months, **how frequently** have you had at least one alcoholic drink?<br>*(READ RESPONSES, USE SHOWCARD)*	Daily 1<br>5-6 days per week 2<br>1-4 days per week 3<br>1-3 days per month 4<br>Less than once a month 5	A2
39	Have you consumed an alcoholic drink within the **past 30 days**?	Yes 1<br>No 2 *If No, go to D1*	A3
40	During the past 30 days, on how many **occasions** did you have at least one alcoholic drink?	Number<br>Don't know 77 └─┴─┘	A4
41	During the past 30 days, when you drank alcohol, **on average**, how many **standard alcoholic drinks** did you have during one drinking occasion?<br>*(USE SHOWCARD)*	Number<br>Don't know 77 └─┴─┘	A5
42	During the past 30 days, what was the **largest number** of standard alcoholic drinks you had on a single occasion, counting all types of alcoholic drinks together?	Largest number<br>Don't Know 77 └─┴─┘	A6
43	During the past 30 days, how many times did you have for **men**: **five or more** for **women**: **four or more** standard alcoholic drinks in a single drinking occasion?	Number of times<br>Don't Know 77 └─┴─┘	A7

EXPANDED: Alcohol Consumption

44	During the past 30 days, when you consumed an alcoholic drink, how often was it with meals? Please do not count snacks.	Usually with meals 1<br>Sometimes with meals 2<br>Rarely with meals 3<br>Never with meals 4	A8
45	During each of the **past 7 days**, how many standard alcoholic drinks did you have each day?<br>*(USE SHOWCARD)*<br>*Don't Know 77*	Monday └─┴─┘	A9a
		Tuesday └─┴─┘	A9b
		Wednesday └─┴─┘	A9c
		Thursday └─┴─┘	A9d
		Friday └─┴─┘	A9e
		Saturday └─┴─┘	A9f
		Sunday └─┴─┘	A9g

CORE: Diet

The next questions ask about the fruits and vegetables that you usually eat. I have a nutrition card here that shows you some examples of local fruits and vegetables. Each picture represents the size of a serving. As you answer these questions please think of a typical week in the last year.

Question		Response	Code
46	In a typical week, on how many days do you **eat fruit**? *(USE SHOWCARD)*	Number of days<br>Don't Know 77 └─┴─┘ *If Zero days, go to D3*	D1
47	How many **servings** of fruit do you eat on **one** of those days? (*USE SHOWCARD)*	Number of servings<br>Don't Know 77 └─┴─┘	D2
48	In a typical week, on how many days do you **eat vegetables**? *(USE SHOWCARD)*	Number of days<br>Don't Know 77 └─┴─┘ *If Zero days, go to D5*	D3
49	How many **servings** of vegetables do you eat on one of those days? *(USE SHOWCARD)*	Number of servings<br>Don't know 77 └─┴─┘	D4

EXPANDED: Diet

50	What type of **oil or fat is most often** used for meal preparation in your household?<br><br>*(USE SHOWCARD)*<br>*(SELECT ONLY ONE)*	Vegetable oil 1<br>Lard or suet 2<br>Butter or ghee 3<br>Margarine 4<br>Other 5 *If Other, go to D5 other*<br>None in particular 6<br>None used 7<br>Don't know 77	D5
		Other └─┴─┴─┴─┴─┴─┴─┘	D5other
51	On average, how many meals per week do you eat that were not prepared at a home? By meal, I mean breakfast, lunch and dinner.	Number<br>Don't know 77 └─┴─┘	D6

CORE: Physical Activity

Next I am going to ask you about the time you spend doing different types of physical activity in a typical week. Please answer these questions even if you do not consider yourself to be a physically active person.
Think first about the time you spend doing work. Think of work as the things that you have to do such as paid or unpaid work, study/training, household chores, harvesting food/crops, fishing or hunting for food, seeking employment. *[Insert other examples if needed].* In answering the following questions 'vigorous-intensity activities' are activities that require hard physical effort and cause large increases in breathing or heart rate, 'moderate-intensity activities' are activities that require moderate physical effort and cause small increases in breathing or heart rate.

	Question	Response	Code
**Work**			
52	Does your work involve vigorous-intensity activity that causes large increases in breathing or heart rate like *[carrying or lifting heavy loads, digging or construction work]* for at least 10 minutes continuously?<br>*[INSERT EXAMPLES] (USE SHOWCARD)*	Yes 1<br>No 2 *If No, go to P 4*	P1
53	In a typical week, on how many days do you do vigorous-intensity activities as part of your work?	Number of days └─┘	P2
54	How much time do you spend doing vigorous-intensity activities at work on a typical day?	Hours : minutes └─┴─┘ : └─┴─┘<br>hrs mins	P3<br>(a-b)
55	Does your work involve moderate-intensity activity, that causes small increases in breathing or heart rate such as brisk walking *[or carrying light loads]* for at least 10 minutes continuously?<br>*[INSERT EXAMPLES] (USE SHOWCARD)*	Yes 1<br>No 2 *If No, go to P 7*	P4
56	In a typical week, on how many days do you do moderate-intensity activities as part of your work?	Number of days └─┘	P5
57	How much time do you spend doing moderate-intensity activities at work on a typical day?	Hours : minutes └─┴─┘ : └─┴─┘<br>hrs mins	P6<br>(a-b)
**Travel to and from places**			
The next questions exclude the physical activities at work that you have already mentioned.<br>Now I would like to ask you about the usual way you travel to and from places. For example to work, for shopping, to market, to place of worship. *[Insert other examples if needed]*			
58	Do you walk or use a bicycle *(pedal cycle)* for at least 10 minutes continuously to get to and from places?	Yes 1<br>No 2 *If No, go to P 10*	P7
59	In a typical week, on how many days do you walk or bicycle for at least 10 minutes continuously to get to and from places?	Number of days └─┘	P8
60	How much time do you spend walking or bicycling for travel on a typical day?	Hours : minutes └─┴─┘ : └─┴─┘<br>hrs mins	P9<br>(a-b)

CORE: Physical Activity, Continued

Question		Response	Code
**Recreational activities**			
The next questions exclude the work and transport activities that you have already mentioned. Now I would like to ask you about sports, fitness and recreational activities (leisure), *[Insert relevant terms]*.			
61	Do you do any vigorous-intensity sports, fitness or recreational *(leisure)* activities that cause large increases in breathing or heart rate like *[running or football]* for at least 10 minutes continuously? *[INSERT EXAMPLES] (USE SHOWCARD)*	Yes 1<br>No 2 *If No, go to P 13*	P10
62	In a typical week, on how many days do you do vigorous-intensity sports, fitness or recreational *(leisure)* activities?	Number of days └─┘	P11
63	How much time do you spend doing vigorous-intensity sports, fitness or recreational activities on a typical day?	Hours : minutes └─┴─┘ : └─┴─┘ hrs mins	P12 (a-b)
64	Do you do any moderate-intensity sports, fitness or recreational *(leisure)* activities that cause a small increase in breathing or heart rate such as brisk walking, *[cycling, swimming, volleyball]* for at least 10 minutes continuously? *[INSERT EXAMPLES] (USE SHOWCARD)*	Yes 1<br>No 2 *If No, go to P16*	P13
65	In a typical week, on how many days do you do moderate-intensity sports, fitness or recreational *(leisure)* activities?	Number of days └─┘	P14
66	How much time do you spend doing moderate-intensity sports, fitness or recreational *(leisure)* activities on a typical day?	Hours : minutes └─┴─┘ : └─┴─┘ hrs mins	P15 (a-b)

EXPANDED: Physical Activity

**Sedentary behaviour**			
The following question is about sitting or reclining at work, at home, getting to and from places, or with friends including time spent sitting at a desk, sitting with friends, traveling in car, bus, train, reading, playing cards or watching television, but do not include time spent sleeping. *[INSERT EXAMPLES] (USE SHOWCARD)*			
67	How much time do you usually spend sitting or reclining on a typical day?	Hours : minutes └─┴─┘ : └─┴─┘ hrs mins	P16 (a-b)

CORE: History of Raised Blood Pressure

Question		Response	Code
68	Have you ever had your blood pressure measured by a doctor or other health worker?	Yes 1<br>No 2 *If No, go to H6*	H1
69	Have you ever been told by a doctor or other health worker that you have raised blood pressure or hypertension?	Yes 1<br>No 2 *If No, go to H6*	H2a
70	Have you been told in the past 12 months?	Yes 1<br>No 2	H2b

EXPANDED: History of Raised Blood Pressure

71	Are you currently receiving any of the following treatments/advice for high blood pressure prescribed by a doctor or other health worker?		
	Drugs (medication) that you have taken in the past two weeks	Yes 1<br>No 2	H3a
	Advice to reduce salt intake	Yes 1<br>No 2	H3b
	Advice or treatment to lose weight	Yes 1<br>No 2	H3c
	Advice or treatment to stop smoking	Yes 1<br>No 2	H3d
	Advice to start or do more exercise	Yes 1<br>No 2	H3e
72	Have you ever seen a traditional healer for raised blood pressure or hypertension?	Yes 1<br>No 2	H4
73	Are you currently taking any herbal or traditional remedy for your raised blood pressure?	Yes 1<br>No 2	H5

CORE: History of Diabetes			
**Question**		**Response**	**Code**
74	Have you ever had your blood sugar measured by a doctor or other health worker?	Yes 1<br>No 2 *If No, go to M1*	H6
75	Have you ever been told by a doctor or other health worker that you have raised blood sugar or diabetes?	Yes 1<br>No 2 *If No, go to M1*	H7a
76	Have you been told in the past 12 months?	Yes 1<br>No 2	H7b

EXPANDED: History of Diabetes			
77	Are you currently receiving any of the following treatments/advice for diabetes prescribed by a doctor or other health worker?		
	Insulin	Yes 1<br>No 2	H8a
	Drugs (medication) that you have taken in the past two weeks	Yes 1<br>No 2	H8b
	Special prescribed diet	Yes 1<br>No 2	H8c
	Advice or treatment to lose weight	Yes 1<br>No 2	H8d
	Advice or treatment to stop smoking	Yes 1<br>No 2	H8e
	Advice to start or do more exercise	Yes 1<br>No 2	H8f
78	Have you ever seen a traditional healer for diabetes or raised blood sugar?	Yes 1<br>No 2	H9
79	Are you currently taking any herbal or traditional remedy for your diabetes?	Yes 1<br>No 2	H10

Participant Identification Number └─┴─┴─┘ └─┴─┴─┘ └─┴─┴─┘

Step 2 Physical Measurements

CORE: Height and Weight			
**Question**		**Response**	**Code**
80	Interviewer ID	└─┴─┴─┘	M1
81	Device IDs for height and weight	Height └─┴─┘<br>Weight └─┴─┘	M2a<br>M2b
82	Height	in Centimetres (cm) └─┴─┴─┘.└─┘	M3
83	Weight<br>*If too large for scale 666.6*	in Kilograms (kg) └─┴─┴─┘.└─┘	M4
84	**For women:** Are you pregnant?	Yes 1 *If Yes, go to M 8*<br>No 2	M5
**CORE: Waist**			
85	Device ID for waist	└─┴─┘	M6
86	Waist circumference	in Centimetres (cm) └─┴─┴─┘.└─┘	M7
**CORE: Blood Pressure**			
87	Interviewer ID	└─┴─┴─┘	M8
88	Device ID for blood pressure	└─┴─┘	M9
89	Cuff size used	Small 1<br>Medium 2<br>Large 3	M10
90	Reading 1	Systolic (mmHg) └─┴─┴─┘	M11a
		Diastolic (mmHg) └─┴─┴─┘	M11b
91	Reading 2	Systolic (mmHg) └─┴─┴─┘	M12a
		Diastolic (mmHg) └─┴─┴─┘	M12b
92	Reading 3	Systolic (mmHg) └─┴─┴─┘	M13a
		Diastolic (mmHg) └─┴─┴─┘	M13b
93	During the past two weeks, have you been treated for raised blood pressure with drugs (medication) prescribed by a doctor or other health worker?	Yes 1<br>No 2	M14

EXPANDED: Hip Circumference and Heart Rate			
94	Hip circumference	in Centimeters (cm) └─┴─┴─┘.└─┘	M15
95	Heart Rate		
	Reading 1	Beats per minute └─┴─┴─┘	M16a
	Reading 2	Beats per minute └─┴─┴─┘	M16b
	Reading 3	Beats per minute └─┴─┴─┘	M16c

Participant Identification Number └─┴─┴─┘ └─┴─┴─┘ └─┴─┴─┘

Step 3 Biochemical Measurements

CORE: Blood Glucose

Question		Response	Code
96	During the past 12 hours have you had anything to eat or drink, other than water?	Yes 1<br>No 2	B1
97	Technician ID	└─┴─┴─┘	B2
98	Device ID	└─┴─┘	B3
99	Time of day blood specimen taken (24 hour clock)	Hours : minutes └─┴─┘ : └─┴─┘<br>hrs mins	B4
100	Fasting blood glucose<br>*Choose accordingly: mmol/l or mg/dl*	mmol/l └─┴─┘ . └─┴─┘<br>mg/dl └─┴─┴─┘ . └─┘	B5
101	Today, have you taken insulin or other drugs (medication) that have been prescribed by a doctor or other health worker for raised blood glucose?	Yes 1<br>No 2	B6

CORE: Blood Lipids

102	Device ID	└─┴─┘	B7
103	Total cholesterol<br>*Choose accordingly: mmol/l or mg/dl*	mmol/l └─┴─┘ . └─┴─┘<br>mg/dl └─┴─┴─┘ . └─┘	B8
104	During the past two weeks, have you been treated for raised cholesterol with drugs (medication) prescribed by a doctor or other health worker?	Yes 1<br>No 2	B9

EXPANDED: Triglycerides and HDL Cholesterol

105	Triglycerides<br>*Choose accordingly: mmol/l or mg/dl*	mmol/l └─┴─┘ . └─┴─┘<br>mg/dl └─┴─┴─┘ . └─┘	B10
106	HDL Cholesterol<br>*Choose accordingly: mmol/l or mg/dl*	mmol/l └─┘ . └─┴─┘<br>mg/dl └─┴─┴─┘ . └─┘	B11

World Health Organization
