# Supplementary material for: Inter-arm difference in systolic blood pressure: Prevalence and associated factors in an African population
Source: PLoS One. 2022 Aug 31;17(8):e0272619. doi: 10.1371/journal.pone.0272619 (PMC9432703; doi:10.1371/journal.pone.0272619)
Supplement: Attachment — Submitted filename: Response to Reviewers.pdf [file pone.0272619.s006.pdf]

Response to Reviewers

Dear editor,

Thank you to giving again the opportunity to review and resubmit our manuscript. We also thank the reviewer for his/her additional comments. A revised version taking into account the reviewer' comments and journal requirements is provided here.

Best regards

PONE-D-21-28514R1

Inter-Arm Difference in Systolic Blood Pressure: Prevalence and Associated Factors in an African Population

PLOS ONE

Journal requirements

Please consider the additional comments of Reviewer 1 in your revision. In addition, could you try to use the inter-arm SBP difference as a continuous variable to investigate the determinants, and perform sensitivity analyses using 15 mm Hg as a cut-off of the large inter-arm BP difference?

We thank the editor for his/her comments. We have responded to the questions of reviewer 1 and conducted a sensitivity analysis using 15 mm Hg as a cut-off of the large inter-arm BP difference as requested.

Regarding suggestion related to the use of inter-arm SBP difference as a continuous variable to investigate the determinants, we observed that all assumptions and conditions for linear regression (e.g., the normality of error distribution and homoscedasticity) were not satisfied. Therefore, we can't run linear regression as our predictions won't be accurate.

Please review your reference list to ensure that it is complete and correct. If you have cited papers that have been retracted, please include the rationale for doing so in the manuscript text, or remove these references and replace them with relevant current references. Any changes to the reference list should be mentioned in the rebuttal letter that accompanies your revised manuscript. If you need to cite a retracted article, indicate the article's retracted status in the References list and also include a citation and full reference for the retraction notice.

Reference list have been checked. No retracted articles have been cited in our work.

Reviewer #1: Thanks for your modification, while there exist minor questions as following.

1. Table 2 and 3 in Result should be capitalized.

We thank reviewer for this comment. We have taken this comment in consideration in the results section.

2. The P value of female in Table 3 is missing.

This information is now provided in the Table 3.

3. You need not to show me the specific process of collinearity, while you need to tell me the final results about the collinearity. Because the backward stepwise method maybe not solid when the independent variables are highly correlated. Furthermore, I wonder why you chose backward instead of stepwise logistic regression?

The stepwise approches includes backward and forward selection.

Backward selection - starting with the full model has the advantage of considering the effects of all variables simultaneously. In contrast with the reviewer statement, this selection approach is especially important when variables in a model are correlated which each other as backward stepwise may be forced to keep them all in the model unlike forward selection where none of them might be entered. In addition, R and other software automatically calculate the best thresholds required to identify the best model (model with the highest likelihood, the one with the lowest Akaike information criterion).

4. In your manuscript, I suppose that the initial model refers to the result of general multiple logistic regression, while the final model refers to the result of backward stepwise selection with the variables which P value less than 0.2 in Initial model. Is my understanding correct? Dose there exist any reference for similar analyses?

The initial model includes the variables which p-value less than 0.2 in univariables analysis. Except, age and sex that we imposed in the final model, the variables in this model are those obtained at the end of the backward stepwise selection. Compared to initial model, the final model was those with the highest likelihood, the one with the lowest Akaike information criterion (Akaike value of initial model is 1434 and which for final model is 1426). Then, in this final model, only the variables which have a p-value less than 0.05 were considered as statistically associated with our dependent variable.

More detail about the backward stepwise selection method are describe in this manuscript “Sanchez-Pinto LN, Venable LR, Fahrenbach J, Churpek MM. Comparison of variable selection methods for clinical

predictive modeling. Int J Med Inform. 2018 Aug;116:10-17. doi: 10.1016/j.ijmedinf.2018.05.006. Epub 2018 May 21. PMID: 29887230; PMCID: PMC6003624. »
