# Supplementary material for: Inter-arm difference in systolic blood pressure: Prevalence and associated factors in an African population
Source: PLoS One. 2022 Aug 31;17(8):e0272619. doi: 10.1371/journal.pone.0272619 (PMC9432703; doi:10.1371/journal.pone.0272619)
Supplement: Attachment — Submitted filename: Response to Reviewers.pdf [file pone.0272619.s007.pdf]

Response to Reviewers

Dear editor,

Thank you to giving again the opportunity to review and resubmit our manuscript. We also thank the reviewer for his/her additional comments. A new revised version taking into account the reviewer' comments is provided here.

Best regards

PONE-D-21-28514R1

Inter-Arm Difference in Systolic Blood Pressure: Prevalence and Associated Factors in an African Population

PLOS ONE

Reviewer #1:

The actual contents of Supplement 1 and 2 are inconsistent with the supposed contents.

We thank reviewer for this comment. Supplement 1 shows the location of the study area on the Benin map . Supplement 2 presents the results from the sensitivity analysis.

The main approaches for stepwise regression are: Forward selection, Backward elimination and Bidirectional elimination (a combination of the above, testing at each step for variables to be included or excluded.), instead of only backward and forward selection. Backward elimination is especially important in case of collinearity, while whether the multivariable model have multicollinearity or not? Please give the Tolerance value and/or VIF value in the results.

Please find below the VIF values of each variable in this model. All VIF were lower than 5 suggesting that there no multicollinearity issues in our final model. As suggested by the reviewer, we added this information in results section in the table 3.

	Age	Gender	Hypertension	Diabetes
VIF	1.11	1.00	1.10	1.00

Please add to the Discussion of your interpretation for the consistent and inconsistent results between sensitivity analyses and the main analyses.

We thank reviewer for this comment. We have taken this comment in consideration in the discussion section. (See Page 10)
