# Supplementary material for: Inter-arm difference in systolic blood pressure: Prevalence and associated factors in an African population
Source: PLoS One. 2022 Aug 31;17(8):e0272619. doi: 10.1371/journal.pone.0272619 (PMC9432703; doi:10.1371/journal.pone.0272619)
Supplement: Attachment — Submitted filename: Reviewer2.docx [file pone.0272619.s005.docx]

Dear editor,

We thank you for giving us the opportunity to revise and resubmit our manuscript. We also thank the reviewers for the helpful comments aiming at improving the article. We propose here a revised version accounting for the comments and suggestions made by the reviewers. We remain available for further request.

Best regards

PONE-D-21-28514

Inter-Arm Difference in Systolic Blood Pressure: Prevalence and Associated Factors in an African Population

PLOS ONE

Comments to the Author

1. Is the manuscript technically sound, and do the data support the conclusions?

The manuscript must describe a technically sound piece of scientific research with data that supports the conclusions. Experiments must have been conducted rigorously, with appropriate controls, replication, and sample sizes. The conclusions must be drawn appropriately based on the data presented.

Reviewer #1: No

Reviewer #2: Yes

2. Has the statistical analysis been performed appropriately and rigorously?

Reviewer #1: No

Reviewer #2: Yes

3. Have the authors made all data underlying the findings in their manuscript fully available?

The PLOS Data policy requires authors to make all data underlying the findings described in their manuscript fully available without restriction, with rare exception (please refer to the Data Availability Statement in the manuscript PDF file). The data should be provided as part of the manuscript or its supporting information, or deposited to a public repository. For example, in addition to summary statistics, the data points behind means, medians and variance measures should be available. If there are restrictions on publicly sharing data—e.g. participant privacy or use of data from a third party—those must be specified.

Reviewer #1: No

Reviewer #2: Yes

4. Is the manuscript presented in an intelligible fashion and written in standard English?

PLOS ONE does not copyedit accepted manuscripts, so the language in submitted articles must be clear, correct, and unambiguous. Any typographical or grammatical errors should be corrected at revision, so please note any specific errors here.

Reviewer #1: Yes

Reviewer #2: Yes

Reviewer reports:

**Reviewer 1:**

1. Prevalence of sIABPD rises in relation to underlying cardiovascular comorbidities of the population studied. Therefore, the prevalence of peripheral arterial disease, CAD, or cerebrovascular disease should be recorded in your manuscript.

We thank the reviewer for these comments. We cannot provide information on peripheral arterial disease because the ankle-brachial index was not measured in 2020. Also, the level of diagnosis of coronary artery disease and cerebrovascular disease is very low due to under-medicalization. However, we provided in table 1 information on participants’ history of cardiovascular and neurological diseases.

2. BP is a variable hemodynamic phenomenon that constantly fluctuates over time, making sequential measurements difficult to compare, therefore, to prevent overestimation and observer bias, sIABPD should be assessed simultaneously at both arms.

Thank you for this comment. We agree with the beat-to-beat variability of BP, but devices measuring simultaneously both arms are not available in practice in Africa, so the clinical interest is questionable. Also, most studies on IABPD have been performed with sequential measurement. This point has been highlighted in the discussion.

3. Lipid related indicators should be included and analyzed as risk factors, such as triacylglycerol, total cholesterol and LDL-C. Moreover, the use of anti-hypertensive drugs, anti-diabetic agents，statins should be recorded and adjusted in your Multivariate analysis.

We have presented all the available variables in Tables 1. However, regarding to the insertion of preventive therapies variable in the multivariate model, this lead a methodological concern. Indeed, there was a strong collinearity between the use of a treatment (e.g. antihypertensive therapy) and presence of factor such as Hypertension, or high blood pressure in the same model. Because the level of use of medication very low in this low income community with low medical resource, we preferred to use the risk factors, rather than the treatment, in the models. The proportion of hypertensive participants on treatment has been reported in the results section.

4. The repeatability of sIABPD is poor, which should be mentioned in the limitation.

We thank reviewer for this comment. We have considered this point in the limitation in discussion section.

5. The results are best to be illustrated in paragraphs:

We have considered this point in the result section

1）Study population
2）Prevalence of sIABP difference
3）Risk factors for sIABP difference
Moreover, the content of the results should be stated more specifically.

We have taken this comment in consideration in the results section.

6. In Statistical analysis, you mentioned that‘The interactions between variables included in multivariable model were tested as well as linearity of quantitative variables’. While, no related results cannot be found in your manuscript.

These analyses are multiple and would dramatically lengthen the results chapter. This kind of analysis is rarely presented in manuscripts. We can send to the editorial team the statistical reports if requested. They have been performed.

7. Your manuscript only excluded pregnant woman, while participants lacking of systolic BP or diastolic BP in both arms or lacking of other risk factors should also be excluded. Moreover, the accurate number or percentage of missing values should be illustrated.

All participants had bilateral measurements and data on risk factors collected.

8. In the results, you mentioned that ‘An increase in the prevalence of sIABPD by age group was observed in both men and women (Figure 3)’. While the P value and P-trend value in Figure 3 should be calculated.

We calculated the p value of the association between sex and sIABPD in each age group.

9. Why the Multivariate analysis included both Initial model and Final model?

Any multivariate analysis with stepwise approach has an initial and then final model. We considered fair to present both so to bring more information. We think that the reader would better understand where we have started and where we have ended by showing both.

Reviewer #2:

1. First, the medical history of subjects was missing in the multiple regression model, especially the history of cardiovascular disease, such as coronary artery disease, stroke or peripheral arteria disease.

We thank the reviewer for this comment. As mentioned earlier, we cannot provide information on peripheral arterial disease because the ankle-brachial index was not measured in 2020. Also, the level of diagnosis of coronary artery disease and cerebrovascular disease is very low due to under-medicalization. However, we provided information on cardiovascular and neurological conditions reported by the participants.

2. Second, the definition of diabetes in this study, which was defined by fasting blood glucose value ≥ 7 mmol/L or currently taking diabetes medication, did not meet the existing diagnostic guidelines.

The definition used for diabetes is those recommended by World Health Organization for STEPS surveys. In addition, the cohort is dynamic, so there are participants who have never been evaluated in previous years. Therefore, we cannot use only the data from previous years to define the history. As we performed single point measurement of blood pressure and glycemia, we have added within the limit section (p 10) the following: “Single point measurement for hypertension and hyperglycemia is also a limitation of our study as it could lead to an overestimation of their prevalence”.

3. In addition, I think that the subjects' lipid levels and their use of lipid-lowering drugs will also be associated with IABPD, which was unfortunately not mentioned in the article.

We agree with the reviewers' comment. However, we do not have data on the participants’ lipid levels or the use of lipid-lowering drugs. We have added this point in the limitation in discussion section.

4. Finally, what is the relationship between different office blood pressure levels and IABPD in this study? Consider including stratified analysis of office blood pressure or as a continuous variable into multiple regression model analysis.

We have not done office BP measurements. Therefore, we cannot determine what the relationship is between the sIABPD and the office BP measurements found in a practice. However, in this new version of the manuscript, we have presented a figure that shows the relationship between the sIABPD and the conventional BP classification groups.
